# A Cross-Provider Healthcare Management Program for Musculoskeletal Disorders: Results of a Randomized Controlled Trial in 22 German Companies

**DOI:** 10.3390/ijerph182211844

**Published:** 2021-11-11

**Authors:** Kyung-Eun (Anna) Choi, Lara Lindert, Lara Schlomann, Christina Samel, Martin Hellmich, Holger Pfaff

**Affiliations:** 1 Center for Health Services Research, Brandenburg Medical School Theodor Fontane, Fehrbelliner Str. 38, 16816 Neuruppin, Germany; lara.lindert@mhb-fontane.de; 2 Institute of Medical Sociology, Health Services Research and Rehabilitation Science, Faculty of Medicine, University Hospital Cologne, Faculty of Human Sciences, University of Cologne, Eupener Str. 129, 50933 Cologne, Germany; lara.schlomann@uk-koeln.de (L.S.); holger.pfaff@uk-koeln.de (H.P.); 3 Institute of Medical Statistics and Computational Biology, Faculty of Medicine, University Hospital Cologne, University of Cologne, Robert-Koch-Str. 10, 50931 Cologne, Germany; christina.samel@uni-koeln.de (C.S.); martin.hellmich@uni-koeln.de (M.H.)

**Keywords:** workplace health promotion, employee health, sick leave, return to work, case management, self-management, early intervention, rehabilitation, reintegration

## Abstract

Musculoskeletal disorders (MSDs) are among the most common cause for reduced work capacity and sick leave. Workplace health promotion programs are often neither tailored to the workplace nor the individual needs of the employees. To counteract lacking intersectional care, this four-year randomized controlled trial (RCT) aimed to investigate the effects of modular coordinating case management (treatment group) compared to supported self-management (control group) on MSD specific sick leave days (routine data), workability (WAI), self-efficacy (self-efficacy scale), and pain (German pain questionnaire, GPQ). The study network comprised 22 companies, 15 company health insurance funds, and 12 pension funds in Germany. Overall, 852 participants (Module A/early intervention: *n* = 651, Module B/rehabilitation: *n* = 190, Module C/reintegration: *n* = 10) participated. Both groups achieved fewer sick leave days, higher workability, and less pain (*p* < 0.0001) at follow-up compared to baseline. At follow-up, the coordinating case management group showed fewer disability days (GPQ), lower disability scores (GPQ), and lower pain intensities (GPQ) than the supported self-management group (*p* < 0.05), but no superiority regarding MSD specific sick leave days, workability, nor self-efficacy. Module A showed more comprehensive differences. The accompanying process evaluation shows barriers and facilitators for the implementation of the program, especially in a RCT setting.

## 1. Introduction

Most industrialized countries face reduced workability due to health problems or disability, resulting in frequent sick leave as an increasing public health problem [1]. For workers, long-term sick leave often leads to increased health risks, aggravation, and chronification of illnesses as well as occupational, social, and economic restrictions [2]. In Germany, musculoskeletal disorders (MSDs) are among the most common cause for sick leave at work. They describe diseases, injuries, and complaints of the musculoskeletal system, often affecting the back or the spine.

Workplace Health Promotion Programs aim to support prevention of MSDs and facilitate return to work. Seeking medical help only in acute phases often means that there has already been a reduced work capacity [3]. Concurrently, most of the coordination programs are neither tailored to the workplace nor the individual needs of the employees. In Germany, many companies cooperate with affiliated health insurance funds that support company health management. These insurance funds often cover a high percentage of the companies’ staff and can therefore facilitate suitable access and the maintaining of health programs considering workplace conditions.

The present study is a four-year randomized controlled trial (RCT) of a modular musculoskeletal health promotion measure. Planned to counteract lacking intersectional MSD care, the study network included 22 German companies (mainly steel and metal manufacturing, automotive industry, trade and service), 12 pension funds, and 15 company health insurance funds with more than 44,000 insured employees in the participating companies. In 2019, insured employees in this system had up to 10,500 sick leave days (per 1000 insured employees) due to MSDs. Illnesses regarding the respiratory system were associated with up to 3500 sick leave days, and psychological diseases caused up to 5190 sick leave days. Moreover, there is an exponential growth in sick leave days for MSD beginning in the age group around 40 years. Furthermore, occupational physicians, test and/or training centers, rehabilitation, and facilities round out the network. Target groups were sick or vulnerable employees with all stages of MSDs. As part of the program, employees were allocated to one of three modules: early intervention (Module A), rehabilitation (Module B), or reintegration (Module C). As a central innovation, a case manager, who led the employees through the whole health care process and involved them in all planning and decision-making processes, was implemented at each site to coordinate intersectional care and act as a communication partner for all involved parties such as test and training centers, OPs, and pension funds. The innovative program was piloted as a single arm observational feasibility study named BeReKo with positive results (“Betriebliches Rehabilitationsprojekt” at Salzgitter AG, Germany).

In the current four-year-trial, a case management concept (treatment group) was tested against supported self-management (control group) as an upgraded version of the current MSD standard care in Germany. We explored whether the coordinating case management compared to supported self-management led to fewer sick leave days, higher workability, less pain, and enhanced self-efficacy in MSD patients. The underlying hypothesis was that a modular, work-place-related treatment supported by case managers addresses individual needs, fosters adherence, and will therefore achieve higher health benefits for employees. Since the study comprised a complex network, the accompanying process evaluation aimed to detect facilitators and barriers of program implementation. The analysis of the qualitative parts were based on a multi-domain approach based on the Consolidated Framework for Implementation Research (CFIR) [4] as well as current project challenges. The CFIR is a conceptual framework to guide systematic assessment of multilevel implementation contexts to identify influencing factors for implementation as well as effectiveness.

## 2. Materials and Methods

**Figure 1:**
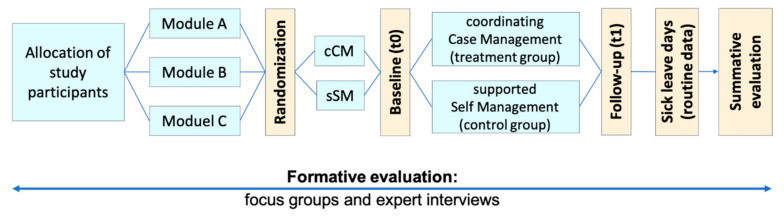
Evaluation design (cCM = coordinating case Management, sSM = supported self-management).

The study (from April/2017 until March 2021) was conducted according to the guidelines of the Declaration of Helsinki, and approved by the University of Cologne’s Faculty of Medicine’s Ethics Commission (project identification code: 17-171). The evaluation concept included a summative/result evaluation in a non-blinded, parallel RCT as well as a formative/process evaluation with focus groups and telephone interviews with key providers (see Figure 1). The RCT adheres to CONSORT guidelines for RCTs [5]. The qualitative parts of the study adheres to the consolidated criteria for reporting qualitative studies (COREQ) guidelines [6].

### 2.1. Summative/Result Evaluation

Depending on their current complaints and illness history, study participants were individually allocated to one of three modules (Module A: early intervention, Module B: rehabilitation, or Module C: reintegration) and then randomized (1:1) into one of two study arms: (1) coordinating case management, or (2) supported self-management (see Figure 1). In the coordinating case management group, study participants received thorough work-related diagnostics and support by the case managers in initiating and maintaining training and/or rehabilitation. Depending on the module, interventions in the treatment group included a 13-week training program adjusted to workplace conditions, an out-patient or in-patient rehabilitation, and psychological assessment for further action (e.g., gradual reintegration). In the supported self-management group, possible interventions were tailored information in regular health care, a Thera-band including instructions for use, possibility to apply for an out-patient or in-patient rehabilitation, and tailored information about possible measures in regular health care for people with severe complaints. For a more detailed description of the modules and study arms, please see earlier publications in [7,8].

Based on the BeReKo data and an estimated effect size of d = 0.3 (with α = 0.05 and β = 0.80), *n* = 175 participants per group were calculated as necessary to detect any statistically significant differences. For randomization, the internet service ALEA (https://nl.tenalea.net/amc/ALEA/Login.aspx, accessed on 10 November 2021) was used. The randomization blocks were stratified for the participating 22 companies to ensure an approximately equal number of both study arm participants at each site. Participants of the supported self-management group were offered to join coordinating case management after completion of the study.

#### 2.1.1. Accessing the Sample

Overall, the participating companies had over 44,000 insured employees in the 15 participating company health insurance funds. The recruitment period took from August 2017 until February 2020. All project partners including the company health insurance funds, pension funds, occupational physicians, companies, and test and training centers delivered information about the project to potential study participants. The main recruiters were case managers. The central project marketing provided templates for recruitment flyers and posters, and website/newsletter information. Each company health insurance fund customized these templates for their own purposes (e.g., some put an additional flyer in the monthly payroll accounting for each employee). Others used office events or general meetings for additional advertisement. The degree of occupational physicians’ integration into recruitment processes varied across sites. They provided a recommendation for module allocation. Afterward, all employees received a consultation with a case manager, who was responsible for the final module assignment based on health records and the occupational physician’s recommendation. Case managers were also responsible for obtaining written informed consent.

#### 2.1.2. Setting Procedure and Data Collection

Case managers’ responsibilities comprised of individually tailored support by initiating work-related diagnostics, fostering cross-sector communication, and being the main contact person for the employees and health providers. As study assistants, they were the main agents for recruitment and coordinated the health care measures.

After the informed consent, study participants were randomized by the case managers. Directly after randomization, the case manager handed out the initial questionnaire (t0) and stamped envelopes for their return to the evaluation institute. The second questionnaire (t1) followed six months later and was also handed out by the case manager. Assessments at test and training centers were documented on separate documentation sheets. These were collected by the case managers and sent to the evaluation institute. Both questionnaires and all documentation sheets had the same pseudonymized identification code to allow matching of the data. The questionnaires and documentation sheets were semi-automatically read in by the Teleform^®^ software and manually checked for consistency.

#### 2.1.3. Primary and Secondary Outcomes

Primary outcomes were subjective work ability measured with the work ability index (WAI) [9] and MSD specific sick leave days. WAI was assessed before (t0) and after the intervention (t1), sick leave days at work in the past six months prior to t0 and after intervention (t1), respectively, were delivered as routine data by the participating company insurance funds. Secondary outcomes were pain (pain intensity (0–100 points) (Mean of current, average, and maximum pain intensity (each on a numeric rating scale from 0–10), multiplied with 10), disability days (0–3 points) (*Within the last three months, on how many days was it difficult for you to continue your daily activities?* (0–3 days = 0 points, 4–7 days = 1 point, 8–15 days = 2 point, >16 days = 3 points)), and disability score (0–3 points) of the German pain questionnaire, GPQ (mean of everyday disability, spare time disability, and workability (each on a numeric rating scale from 0–10), multiplied with 10), then classified according to: 0–29 = 0 points, 30–49 = 1 point, 50–69 = 2 points, >70 = 3 points [10], and self-efficacy [11]. The secondary outcomes were also assessed with the t0 and t1 questionnaires. Additional exploratory outcomes encompassed further standardized measures as EFL-tests (evaluation of functional capacity) [12] and PACT (Performance Assessment and Capacity Training) [13] (both for the coordinating case management group in Modules A and C only), but also self-developed questions regarding general patient satisfaction with the study, interaction with the case manager, and lifestyle.

#### 2.1.4. Data Analysis

The data analysis was based on the intention-to-treat-principle. All outcomes were tested for normal distribution in the overall study sample. Due to the outcomes’ characteristics, all comparisons in means were performed non-parametrically with the Mann–Whitney U test or Wilcoxon tests at a significance level of α = 0.05. Additional exploratory analyses were carried out for Module A. Further Module B and C analyses have been left out due to the size of the subsamples and have been evaluated descriptively. As all analyses are exploratory, no adjustment for multiple testing was made. All statistical analyses were carried out with IBM^®^ SPSS Statistics 27. 

### 2.2. Formative/Process Evaluation

To evaluate the implementation process and to identify facilitators and barriers from different perspectives, we repeatedly interviewed case managers, occupational physicians, and staff of fitness/training/test centers from March 2018 until September 2020. All study participants gave their informed consent.

#### 2.2.1. Accessing the Sample

All case managers, occupational physicians, and staff of fitness/training/test centers with duties in the RCT were invited to share their experiences and views on the implementation in either focus groups (case managers) or via telephone interviews (occupational physicians and staff of fitness/training/test centers). For the case managers, focus groups were chosen as a method to stimulate the exchange of experiences and developed strategies between the case managers. The topic lists were designed to stimulate discussion about aspects that might not emerge in individual telephone interviews.

#### 2.2.2. Setting Procedure and Data Collection

All topic lists aimed at discovering barriers and facilitators of program implementation. Due to the specific tasks and duties for each occupation, the guides were adapted to their responsibilities and current developments in the study. The semi-structured guides were designed by the research team by considering major aspects of the Consolidated Framework for Implementation Research (CFIR) [4] as well as current project challenges, assessed via feedback in project meetings of the wider network. Since the implementation afforded the interplay of different parties within the network, we used a multi-domain framework that accounts for different perspectives that has been widely used in implementation studies [14]. All guides were pretested. Potential interviewees were approached by members of the study team. Participants were familiar with the interviewer/moderator of focus groups from previous project meetings. In each focus group, one research assistant took notes. In the individual telephone interviews, field notes were made by the interviewer. Interviewers/moderator and research assistants, as part of the evaluating institute, had interdisciplinary backgrounds, prior experience with qualitative research, and were all female researchers. The telephone interviews were collected at the workplace, while focus group locations were specifically organized at one of the company sites. During the data collection, no one else was present.

#### 2.2.3. Data Analysis

All focus groups and telephone interviews were audio recorded, verbatim transcribed, and pseudonymized. Three researchers, consisting of K.-E.C. (Anna) (psychologist, Ph.D.), L.S. (health economist, M.A.), and L.L. (rehabilitation scientist, M.A.), coded the data using MAXQDA 2020. The use of field notes and several rounds of peer review ensured intersubjectivity of the results and minimized possible bias in data analysis. Data analysis followed a deductive-inductive approach. Category schemes were deductively-inductively driven and considered components of the Consolidated Framework for Implementation Research (CFIR) [4]. Inductively developed categories were discussed and added to the coding scheme in an iterative procedure. Changes to the coding scheme were repeatedly discussed and reviewed within the research team.

Initially, one interview was coded by K.-E.C. (Anna), L.S., and L.L. in teamwork to check mutual understanding of the coding scheme and the overlapping of codes. Any disagreements were solved through discussion and reflection. Afterward, the remaining material was independently coded by the study team members. Finally, L.S. repeated the coding process for all transcripts to ensure intersubjective agreement. All coders discussed and agreed on data interpretation. Initially, quotes to each category were summarized in tables. Quote representative were selected to reflect (potential) different valences within each category. For categories with diverging perspectives, several quotes were selected to achieve a more comprehensive overview. The selection process involved discussion and reflection. All coders agreed on selected quotes. Finally, quotes representative for the findings were selected, and translated from German to English. Study participants had the opportunity to comment or correct transcripts, but none provided feedback.

## 3. Results

### 3.1. Summative/Result Evaluation

#### 3.1.1. Sample

**Figure 2:**
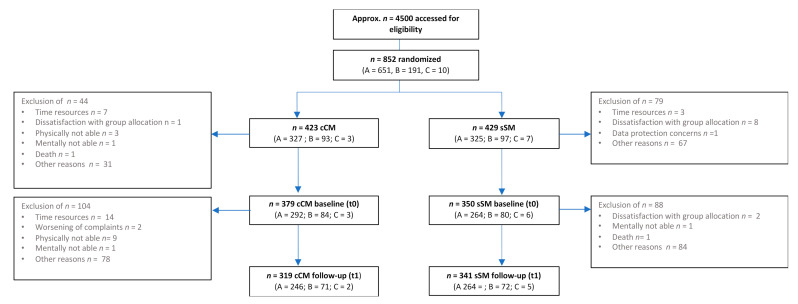
Study flow chart.

Overall, 852 participants (Module A: *n* = 651, Module B: *n* = 190, Module C: *n* = 10) were randomized (see Figure 2). Of these, 89.5% returned t0-questionnaires and 71.4% returned t1-questionnaires. Matched questionnaires (t0 and t1) were present for 69.5%.

The age and gender distribution reflects the percentages of MSD concerned employees in the participating insurance funds and were comparably distributed in the coordinating case management and supported self-management group (coordinating case management: 289 male, 86 female, four not stated; supported self-management: 273 male, 77 female). Most of the study participants were male and aged between 40 and 60 years (coordinating case management: *n* = 17 under 29 years, *n* = 42 between 30 and 39 years, *n* = 100 between 40 and 49 years, *n* = 187 between 50 and 59 years, *n* = 33 between 60 and 69 years; supported self-management: *n* = 20 under 29 years, *n* = 43 between 30 and 39 years, *n* = 99 between 40 and 49 years, *n* = 170 between 50 and 59 years, *n* = 19 between 60 and 69 years). During the trial, two study participants naturally died, two reported worsening of complaints, 12 reported to be physically not able to continue, three were reported to be mentally not able to continue. These observed events were judged to be not in direct connection with the study interventions by case managers and/or the occupational physician. Eleven study participants reported to be dissatisfied with the study arm allocation and dropped out of the study.

#### 3.1.2. Overall

As expected, there were no group differences concerning the primary and secondary outcomes at baseline (see Table 1). At follow-up, coordinating case management group showed fewer disability days (German pain questionnaire), lower disability scores (German pain questionnaire), and lower pain intensities (German pain questionnaire) than the supported self-management group (control group). However, there were no group differences regarding sick leave days, work ability, nor self-efficacy. Nevertheless, both coordinating case management group and supported self-management group significantly improved regarding all outcomes except that of self-efficacy from baseline to follow-up (see Table 2).

#### 3.1.3. Module A

The most improvements were found for Module A, which was the module with the highest number of participants.

##### Primary and Secondary Outcomes

In Module A, the coordinating case management group had fewer disability days (GPQ), lower disability scores (GPQ) and lower pain intensities (GPQ), higher self-efficacy values as well as higher work ability (WAI) at follow-up than the supported self-management group (see Table 3). However, there were no group differences regarding MSD specific sick leave days. Similar effects were found in the timepoint analyses; except of self-efficacy, all outcomes showed health benefits for both groups (see Table 4).

##### EFL, BMI, Pain VAS, PACT

The positive effects in Module A’s coordinating case management group were underlined by EFL tests, PACT, BMI (body mass index), and pain VAS (visual analogue scale). Of the 123 study participants, EFL endurance slightly worsened in 14, remained unchanged in 20, slightly improved in 51, and strongly improved in 38. Approximately 72.4% yielded at least a slight improvement in endurance. Of the 112 study participants, EFL coordination slightly worsened in one, remained unchanged in 24, slightly improved in 17, and strongly improved in 17. Approximately 77.7% achieved at least a slight improvement in coordination. Of the 113 study participants, EFL mobility slightly worsened in three, remained unchanged in 13, slightly improved in 62, and strongly improved in 35. Thus, approximately 85.9% showed a slight improvement in mobility. Of the 127 study participants, EFL power slightly worsened in eight, remained unchanged in five, slightly improved in 46, and strongly improved in 68. Thus, approximately 89.7% showed at least a slight improvement in power. Members of the coordinating case management group in Module A reduced their mean BMI from 27.41 at baseline to 27.06 at follow-up (*p* = 0.009), reduced mean pain VAS from 3.59 to 2.12 (*p* = 0.000), and increased their PACT score from 154.07 to 166.21 (*p* = 0.000).

### 3.2. Formative/Process Evaluation

The process evaluation took place in three waves, each one in 2018, 2019, and 2020. The eight semi-structured focus groups with case managers took about two to three hours each, and the participation rate was high (2018 *n* = 16, 2019 *n* = 13, 2020 *n* = 15). The telephone interviews with occupational physicians lasted approximately 20 to 40 min each (2018 *n* = 9, 2019 *n* = 13), and the inteviews with staff of the fitness/training/test centers lasted approximately 20 to 60 min each (2020 *n* = 9).

**Figure 3:**
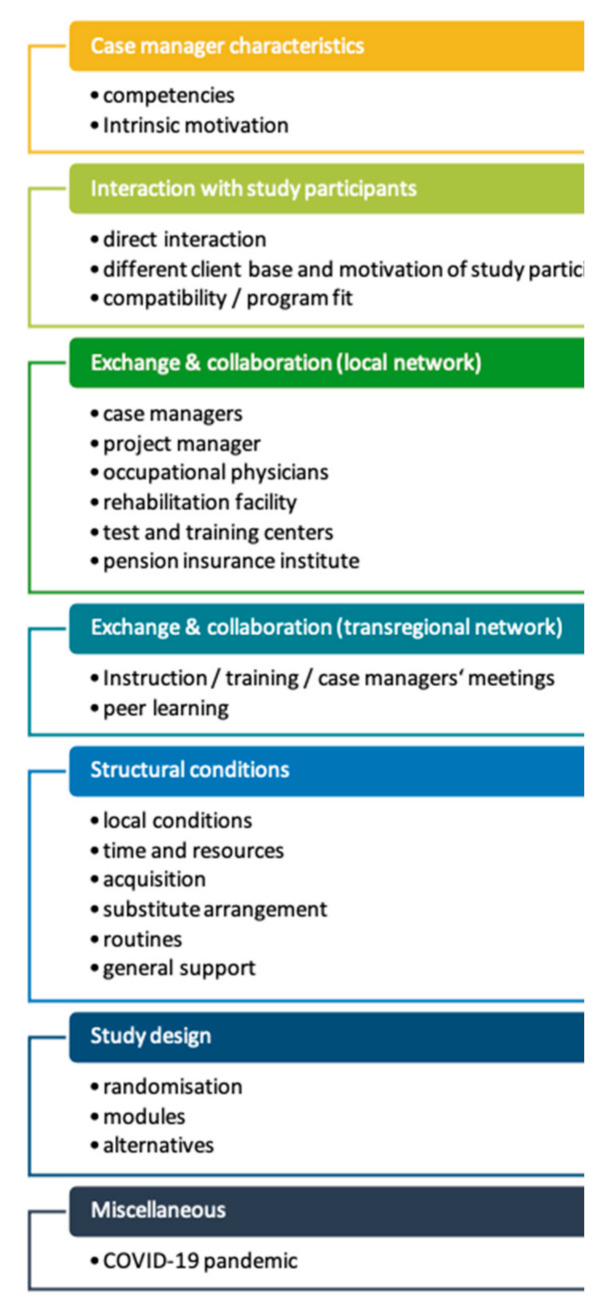
Implementation barriers and facilitators.

The main implemenentation facilitators and barriers are listed in Figure 3. Please see other publications for deeper sub-analyses [7,8].

A central component of the coordinating case management treatment group is the case manager. Which competencies should a good case manager have? In the focus groups, the following aspects were reported: good communication skills, empathy, proper time management, the capacity to building a trustful interaction, and—especially with limited time resources—a high level of intrinsic motivation.

“*Well, good communication skills are essential, proper empathy… to connect with others in their specific circumstances, not only concerning their diseases, but sometimes also their social environment. And you should a good time management—that’s really important*”.

(focus group with case managers)

“*Within the last two months, I’ve been on my own for four weeks. The work load is too high. (…) If someone who is interested in the study approaches me, then okay… we can meet and talk, but apart from that (…) I’ll leave it*”.

(focus group with case managers)

Personal contact was very important to build a good relationship with the study participants, particularly if they had other expectations regarding the study treatments. Occupational physicians and case managers observed various forms of motivation within the study participants to adhere to the program. Reported challenges include shift work and business trips. Test and training centers with flexible opening hours facilitated the access.

“*I sometimes almost feel personally related to our employees and I know a lot of them by name… I think, in this company, that’s a big advantage for me. They have a high level of trust in me*”.

(focus group with case managers)

“*I can probably get office people to do something for their health two times a week faster than people working on the assembly line for nine hours, who are really tired in the evening […]. But there are also motivated people on the assembly line who say: ‘Nah, that is important to me and that is why I invest the time*’ ”.

(telephone interview with occupational physician)

“*Well, it’s difficult, since most of the employees cannot complete the full training program because of regular business trips*“.

(focus group with case managers)

Peer-learning was a main facilitator reported by all key providers. Special trainings for the study staff ensured regional and cross regional exchange of experiences and strategies. However, study staff that missed these trainings had problems to catch up with their colleagues and/or intensify the network contacts, especially if they worked part-time.

“*Learning by doing and feedback—that’s always helpful!*”.

(focus group with case managers)

“*Yes, I had contact to other occupational physicians of the project. Everything works fine, also with the other network partners: the test and training center, for example,… they have an important interface function for us*”.

(telephone interview with occupational physician)

“*Missed meetings are a big issue. Our occupational physician did not attend the regional training. That caused a strange feeling*“.

(focus group with case managers)

Main barriers were alternative offers, long distances between sites, a lack of routines, and in some cases, a lack of suitable rooms. Additionally, the study design was experienced as challenging.

“*We have so many other early prevention courses, gymnastics and so on… that means they can choose whatever from a huge range—no matter, if there’re in the self-management group*”.

(focus group with case managers)

“*It was off to a slow start, … advertisement could have been better, but now we’ve informed all employees and that made it a lot better*”.

(focus group with case managers)

Restriction due to the COVID-19 pandemic hindered some trainings, and had negative consequences for the motivation of study participants. Home office regulations led to a loss in personal contacts. Overall, limitations due to the pandemic were rated as moderate, since the active project phase was almost at the end. Marketing support was rated positively and as helpful.

“*I had the support of our occupational physician, also from the personnel department. (…) But since the appearance of the pandemic, it’s all over. Most of them work from home… and we have short-time work now—it is a ghost town*”.

(focus group with case managers)

## 4. Discussion

Based on the encouraging results of the BeReKo pilot trial, the aim of the current study was to assess the effect of an innovative modular, work-place-related, coordinated treatment (coordinating case management) compared to an extended standard care procedure (supported self-management) with the support of a network of company health insurance funds. The underlying hypothesis was that a treatment that addresses individual needs via case managers’ support and specific work-related training will show superiority, since it will be easy to connect to and therefore fosters the adherence of study participants.

The recruited study population of the RCT reflects the age, gender, and ill severity distribution of employees with MSD in the participating companies. Drop-out rates were moderate, and no (serious) adverse events in connection with the interventions were observed. Both coordinating case management as well as the standard care control group (supported self-management) yielded better outcomes from baseline to follow-up. Overall, the hypotheses concerning group differences in workability, MSD specific sick leave days, and self-efficacy could not be verified. The coordinating case management group showed better results only for pain (GPQ). However, in Module A, the found differences may support the idea that case management for this group achieves more comprehensive health benefits.

According to a review by van Vilsteren et al. [15], workplace interventions can significantly improve time to first and lasting return-to-work with MSD patients more than usual care. Pain as well as functional status benefits from such interventions [15]. To limit long-term sick leave, return-to-work coordination programs in workers on sick leave, however, yield comparably small benefits [16]. Multi-domain interventions are asked for [17]. The setting around company health insurance funds seemed to be particularly promising, since they have a high percentage of insured employees in the participating companies and therefore possess specific knowledge about work-place related requirements. Moreover, most of the case managers could fall back on an existing fundamental cooperation network. Successful case managers are characterized by finding a balance between the accompanying key functions and tasks: advocacy, care coordination, case monitoring and patient needs assessment, community engagement, education, administration and research activities, psychosocial support, navigation of services, and reduction in barriers to care [18]. To assure a seamless transition into standard care after a positive evaluation of the program, case managers already implemented in the real-world setting were also selected to serve as study case managers. This facilitated the building of the regional network, but also caused challenges due to the randomization of the non-blindable treatments that might have had a negative impact on study implementation [8]. Study participants of the supported self-management were offered to take part in a comparable coordinating case management measure after completion of the study, but the waiting time seemed to have been too long. Based on their strong personal relationship with the insured employees, some case managers perceived conflicts with the “inferior” supported self-management group and offered particularly attentive advice. In a real-world setting, a single consultation, as designed here in supported self-management to support standard care, will probably not have the same effects.

The case management concept is a safe and valued concept by key providers. Considering the efforts and costs accompanying its implementation, the found effects do not support the idea of a clear superiority to a supported self-management concept. Only coordinating case management in Module A had a superior performance compared to the supported self-management (except sick leave days). Nevertheless, many company insurance funds have already declared continuing the coordinating case management concept as part of their catalogue of services.

### Strengths and Limitations

A major strength of the study is the participation of all key providers from the workplace, test and training centers, rehabilitation sites, pension funds, and company health insurances. The created network will also facilitate the local and regional cooperation within study partners in the management for other diseases. Training concepts, marketing materials, and study material will be useable with minor changes.

Although the main analyses were sufficiently powered and allowed for sub-analyses in Module A, a full analytic model considering intervention, module, and time effects was not carried out. The necessary sample size for such a model would have been much higher. Moreover, the overall recruitment rates in this study were already low despite the huge target group. Additional study participants would have allowed for further sub-analyses to specify treatment effects, (e.g., modular concept, work-related training effects). A main barrier for recruitment reported by the case managers was randomization on the individual level [8]. However, the majority of the participating partners would not have agreed to a cluster-randomized design.

The nature of the evaluation design with a formative and summative approach delivered further insight into the implementation mechanisms and valuable information treatment effects.

## 5. Conclusions

Overall, the case management concept was not convincingly superior to supported self-management. Valuable alternatives already seem to be available in the service catalogue of the company insurance funds as part of their standard care. The most promising results were found in the early prevention group (Module A). Considering additional expenses, researchers and politicians could use the obtained results regarding the implementation of the study program to design new studies and health services.

## Figures and Tables

**Table 1:** Group differences’ analyses: coordinating case management (cCM) vs. supported self-management (sSM) at baseline (t0) and follow-up (t1) (* = *p* < 0.05, ** = *p* < 0.01).

	Meanc cCM/sSM	Percentiles			*n* cCM/sSM	*p*-Value	r-Value
		25. cCM/sSM	50. (Median) cCM/sSM	75. cCM/sSM			
t0 disability days	2/2	0.00/0.00	2.00/2.00	3.00/3.00	363/335	0.955	0.0022
t1 disability days	1/1	0.00/0.00	0.00/1.00	2.00/2.00	256/248	0.041 *	0.091
t0 disability score	47.89/46.67	30.00/26.67	50.00/46.67	66.67/66.67	379/347	0.498	0.0252
t1 disability score	31.17/36.15	10.00/10.00	20.00/33.33	50.00/56.67	268/262	0.030 *	0.0942
t0 pain intensity	54.56/54.13	43.33/40.00	56.67/56.67	70.00/70.00	378/350	0.931	0.0032
t1 pain intensity	40.40/46.19	23.33/23.33	40.00/46.67	56.67/66.67	264/259	0.005 **	0.1233
t0 self-efficacy	29.92/30.06	28.00/28.00	30.00/30.00	32.00/32.00	375/339	0.923	0.0036
t1 self-efficacy	30.14/29.89	28.00/28.00	30.00/30.00	32.00/32.00	265/255	0.146	0.0637
t0 work ability	24.29/24.42	20.00/21.00	24.00/24.50	29.00/29.25	375/345	0.679	0.0154
t1 work ability	26.73/25.75	22.00/22.00	27.50/26.50	32.00/31.00	266/254	0.088	0.0748
t0 sick leave days (MSD specific)	29/28	3.00/4.25	12.00/14.00	33.00/33.75	163/200	0.804	0.013
t1 sick leave days (MSD specific)	18/13	0.00/0.00	2.00/0.00	19.00/16.75	163/200	0.376	0.0465

**Table 2:** Timepoint differences’ analyses: baseline (t0) vs. follow-up (t1), separately for coordinating case management (cCM) and supported self-management (sSM) (*** = *p* < 0.001).

Over All Modules		Mean t0/t1	Percentiles			*n*	*p*-Value	r-Value
			25. t0/t1	50. (Median) t0/t1	75. t0/t1			
**Case Management**	disability days	2/1	0.00/0.00	2.00/0.00	3.00/2.00	243	0.000 ***	0.4806
	disability score	45.20/30.75	26.67/10.00	46.67/30.00	63.33/47.50	262	0.000 ***	0.547
	pain intensity	53.00/40.15	40.00/23.33	53.33/40.00	66.67/56.67	259	0.000 ***	0.5673
	sick leave days (MSD-specific)	29/18	3.00/0.00	12.00/2.00	33.00/19.00	163	0.000 ***	0.2953
	self-efficacy	29.88/30.17	28.00/29.00	30.00/30.00	32.00/32.00	256	0.191	0.0818
	work ability	24.78/26.83	20.88/22.38	25.00/27.50	29.63/32.00	258	0.000 ***	0.3917
**Self Management**	disability days	2/1	0.00/0.00	2.00/1.00	3.00/2.00	230	0.000 ***	0.3809
	disability score	46.85/35.88	26.67/10.00	46.67/33.33	70.00/56.67	249	0.000 ***	0.4136
	pain intensity	54.00/45.93	40.00/23.33	56.67/46.67	70.00/66.67	249	0.000 ***	0.3913
	sick leave days (MSD-specific)	28/13	4.25/0.00	14.00/0.00	33.75/16.75	200	0.000 ***	0.3971
	self-efficacy	30.29/29.95	29.00/28.00	30.00/30.00	32.00/32.00	241	0.148	0.0933
	work ability	24.69/25.79	21.00/22.00	25.00/26.50	29.50/31.00	240	0.000 ***	0.2299

**Table 3:** Group differences’ analyses in Module A: coordinating case management (cCM) vs. supported self-management (sSM) at baseline (t0) and follow-up (t1) (* = *p* < 0.05, ** = *p* < 0.01).

	Mean cCM/sSM	Percentiles			*n* cCM/sSM	*p*-Value	r-Value
		25. cCM/sSM	50. (Median) cCM/sSM	75. cCM/sSM			
t0 disability days	1/1	0.00/0.00	2.00/2.00	3.00/3.00	284/253	0.898	0.0055
t1 disability days	1/1	0.00/0.00	0.00/0.00	1.00/2.00	200/191	0.003 **	0.1507
t0 disability score	43.38/42.50	26.67/20.83	43.33/40.00	60.00/63.33	293/260	0.602	0.0222
t1 disability score	26.36/32.99	8.33/10.00	20.00/30.00	43.33/53.33	209/203	0.012 *	0.1241
t0 pain intensity	51.60/50.52	40.00/36.67	53.33/53.33	65.00/66.67	293/263	0.759	0.013
t1 pain intensity	36.72/43.50	20.00/23.33	36.67/43.33	50.00/63.33	206/201	0.004 **	0.1447
t0 self-efficacy	30.16/30.09	28.00/28.00	30.00/30.00	33.00/32.00	291/256	0.503	0.0286
t1 self-efficacy	30.52/30.06	29.00/28.00	31.00/30.00	33.00/32.75	208/200	0.049 *	0.0976
t0 work ability	25.60/25.64	22.00/22.0	26.00/26.00	30.00/30.00	291/259	0.888	0.006
t1 work ability	27.93/26.57	24.00/22.50	28.50/27.50	32.00/32.00	208/197	0.035 *	0.105
t0 sick leave days (MSD specific)	24/22	3.00/1.75	9.00/12.00	22.50/22.25	105/138	0.641	0.03
t1 sick leave days (MSD specific)	11/12	0.00/0.00	0.00/0.00	15.00/13.00	105/138	0.786	0.0174

**Table 4:** Timepoint differences’ analyses in Module A: baseline (t0) vs. follow-up (t1), separately for coordinating case management (cCM) and supported self-management (sSM) (* = *p* < 0.05, ** = *p* < 0.01, *** = *p* < 0.001). (NRS = numeric rating scale, VAS = visual analogue scale).

		Mean t0/t1	Percentiles			*n*	*p*-Value	r-Value
			25. t0/t1	50. (Median) t0/t1	75. t0/t1			
**Case management**	disability days	1/1	0.00/0.00	1.00/0.00	2.00/1.00	192	0.000 ***	0.4858
	disability score	40.67/26.11	20.00/6.67	40.00/20.00	56.67/41.67	205	0.000 ***	0.6151
	pain intensity (NRS)	50.02/36.65	36.67/20.00	50.00/36.67	63.33/50.00	203	0.000 ***	0.5665
	sick leave days (MSD-specific)	24/11	3.00/0.00	9.00/0.00	22.50/15.00	105	0.000 ***	0.385
	self-efficacy	30.05/30.55	28.00/29.00	30.00/31.00	33.00/33.00	203	0.033 *	0.1499
	work ability	26.13/27.95	22.50/24.00	26.50/28.50	30.00/32.00	203	0.000 ***	0.3644
	BMI	27.41/27.06	24.25/23.80	26.80/26.10	29.30/29.53	133	0.009 **	0.2255
	pain (VAS)	3.59/2.12	2.00/0.00	3.00/2.00	5.00/3.00	134	0.000 ***	0.6164
	PACT	154.07/166.21	138.00/154.00	156.00/171.00	178.00/188.00	133	0.000 ***	0.5186
**Self management**	disability days	2/1	0.00/0.00	2.00/1.00	3.00/2.00	179	0.000 ***	0.3351
	disability score	42.65/32.97	20.00/10.00	40.00/30.00	63.33/53.33	195	0.000 ***	0.3593
	pain intensity	50.39/43.47	36.67/23.33	53.33/43.33	66.67/63.33	196	0.000 ***	0.3418
	sick leave days (MSD-specific)	22/12	1.75/0.00	12.00/0.00	22.25/13.00	138	0.000 ***	0.3333
	self-efficacy	30.22/30.09	29.00/28.00	30.00/30.00	32.00/33.00	191	0.717	0.0262
	work ability	25.93/26.55	22.00/22.50	26.00/27.25	30.00/31.88	188	0.032 *	0.1561

## Data Availability

The datasets used and/or analyzed during the current study are available from the study group on reasonable request. Please contact the corresponding author.

## Abbreviations

BeReKo	“*Betriebliches Rehabilitationsprojekt”* (observational pilot study)
BMI	Body mass index
EFL	“Evaluation funktioneller Leistungsfähigkeit” (evaluation of functional capacity)
GPI	German pain index
MSD	Musculoskeletal disorder(s)
PACT	Performance Assessment and Capacity Training
RCT	Randomized controlled trial
VAS	Visual analogue scale
